# A Rare Pathology Mimicking the Gallstone: Heterotopic Pancreas in the Gallbladder

**DOI:** 10.7759/cureus.2659

**Published:** 2018-05-21

**Authors:** Murat F Ferhatoglu, Taner Kivilcim, Abdulcabbar Kartal, Ali I Filiz

**Affiliations:** 1 General Surgery, Okan University Medical Faculty, Istanbul, TUR

**Keywords:** cholecystopaty, gallbladder, heterotropic pancreas, ectopic pancreas

## Abstract

The placement of pancreatic tissue in an organ outside the pancreas is called pancreatic heterotopy. Heterotopic pancreatic (HP) tissue is frequently observed in the stomach and duodenum, while the gallbladder is an extremely rare localization. In this article, we present pancreatic heterotopy located in the gallbladder, a rarely observed embryologic anomaly, with the study of two cases and a review of the literature.

## Introduction

Apart from normal pancreatic tissue or independently of ductus-vascular structures associated with the pancreas, the appearance of pancreatic tissue in another organ is termed pancreatic heterotopy. The first heterotopic pancreatic tissue was described in 1729 by Jean Schultz in the ileal diverticulum and, in 1859, two cases of histologically proven heterotopic pancreatic (HP) tissue were presented by Klob. The first HP case located in the gallbladder was presented by Otschkin in the beginning of the twentieth century [1]. When the literature was examined, it was seen that there are 34 case reports since 1916. In this article, we present pancreatic heterotopy located in the gallbladder, a rarely observed embryologic anomaly, with the study of two cases and a review of the literature.

## Case presentation

Case 1

**Figure 1:**
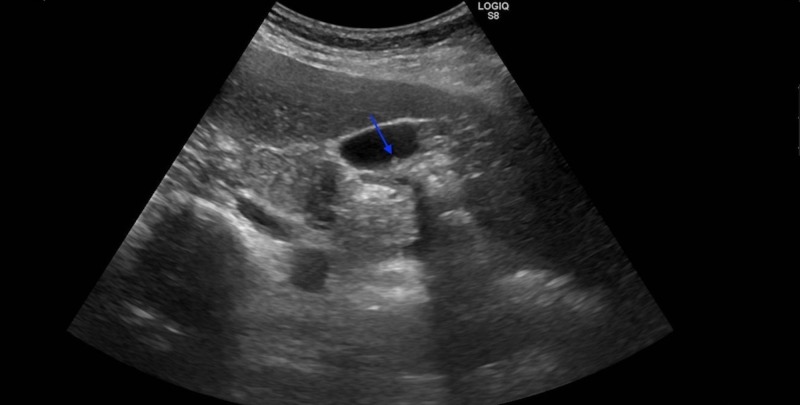
Ultrasonographic image of the polyp (blue arrow)

**Figure 2:**
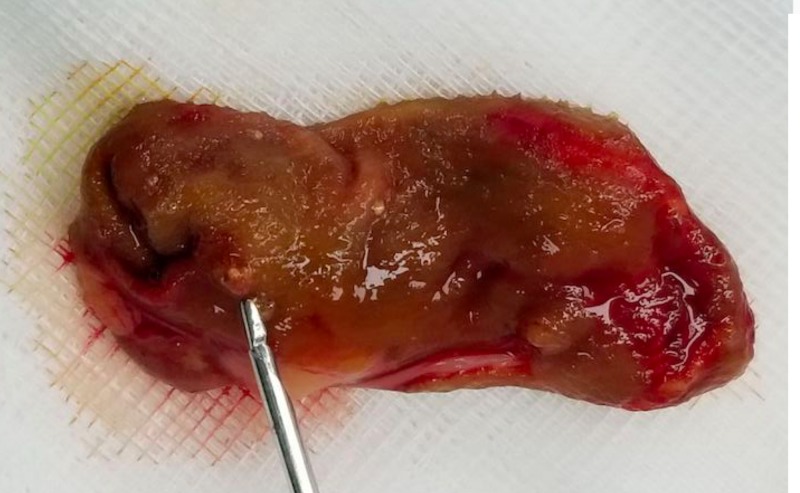
Needle shows a polyp in the gallbladder

**Figure 3:**
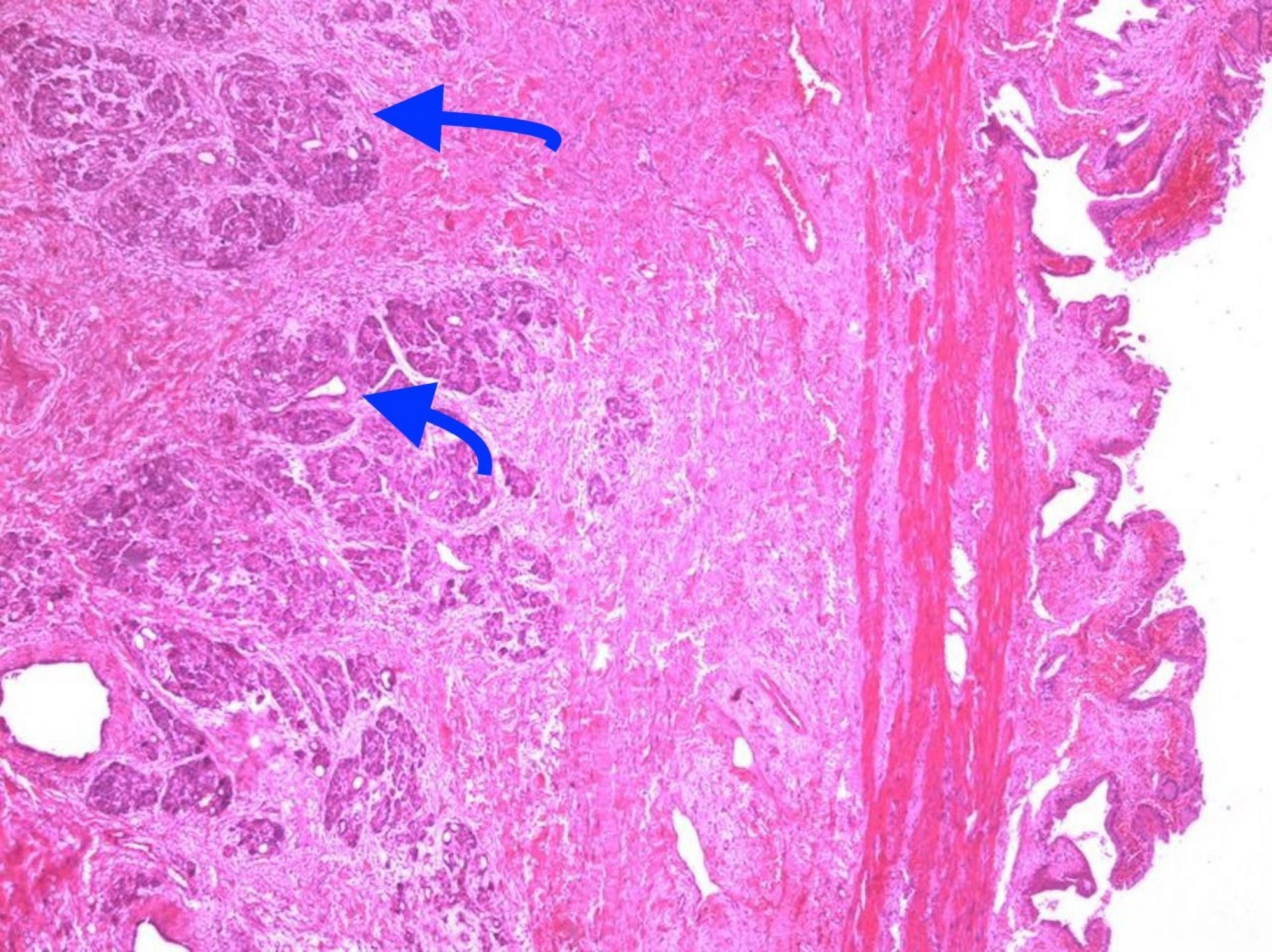
Heterotopic pancreatic tissue located in gallbladder (H&E x100) H&E: hematoxylin and eosin

A 48-year-old female patient presented to the surgery clinic with complaints of three years of epigastric pain, which had increased for the last six months, and nausea after eating fatty foods. A complete blood count revealed a white blood count of 9550 cells/mm^3^ (4600–10200 cells/mm^3^) and a hematocrit level of 38% (40%–54% ). Her electrolytes, liver function tests, blood urea nitrogen, and creatinine were normal. A 7-mm polyp in the gallbladder was detected (Figure 1) in the abdominal ultrasonography of the patient with a normal physical examination and no known disease. The patient underwent a laparoscopic cholecystectomy and was discharged on the first postoperative day, uneventfully. A pathologic examination of the specimen revealed an 8-mm polyp (Figure 2), including mononuclear cell infiltration consistent with chronic cholecystitis, thickening in the gallbladder wall, fibrosis, and 6x4 mm heterotopic pancreatic tissue located in the submucosal area of the fundus (Figure 3). No further complications occurred in the three-month follow-up of the patient. 

Case 2

**Figure 4:**
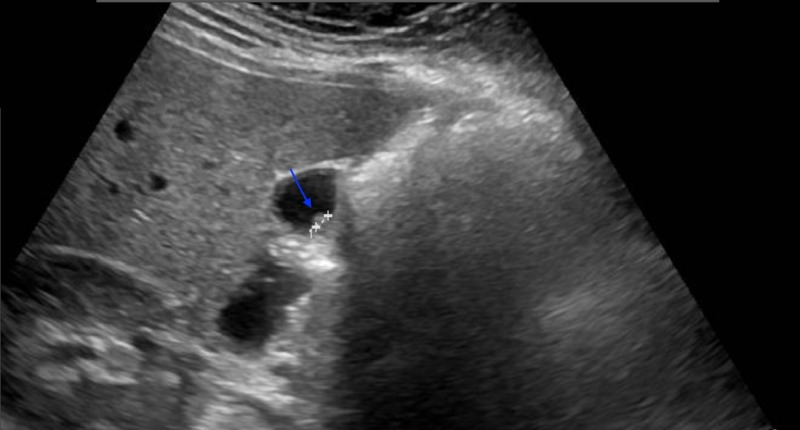
Ultrasonographic image of the polyp (blue arrow)

**Figure 5:**
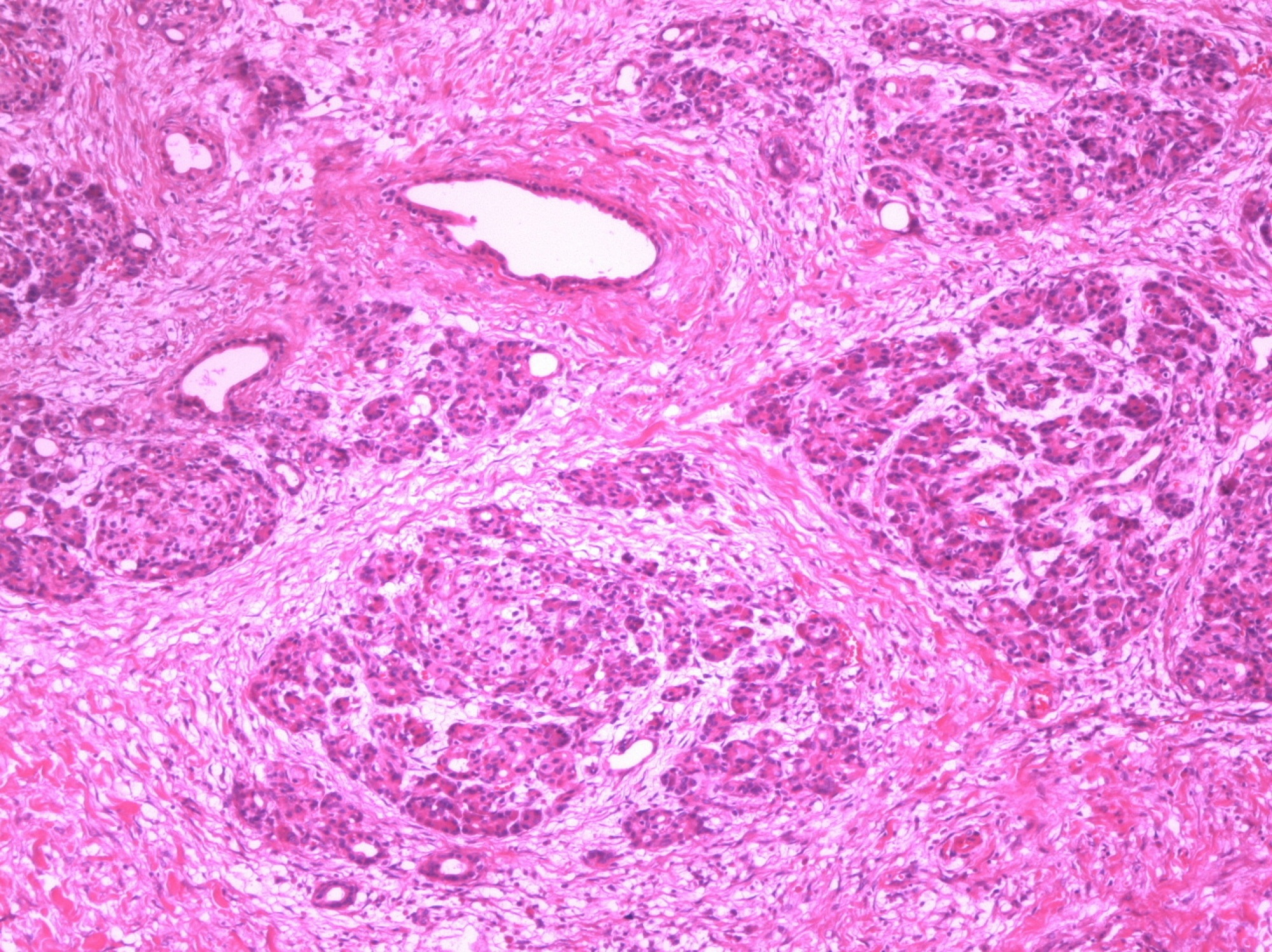
Heterotopic pancreatic tissue located in gallbladder (H&E x100) H&E: hematoxylin and eosin

A 39-year-old male patient presented to the surgery clinic with a one-year history of right upper quadrant pain. The complete blood count revealed a white blood count of 7200 cells/mm^3^ (4600–10200 cells/mm^3^) and a hematocrit level of 45% (40%–54% ). His electrolytes, liver function tests, blood urea nitrogen, and creatinine were normal. A 6-mm polyp and sludge were detected within the gallbladder in the abdominal ultrasonography of the patient (Figure 4) with a normal physical examination and no known disease. The patient underwent laparoscopic cholecystectomy and was discharged on the first postoperative day uneventfully. A pathologic examination revealed sludge, mononuclear cell infiltration consistent with chronic cholecystitis, thickening in the gallbladder wall, fibrosis, and 7-mm heterotopic pancreatic tissue in the gallbladder corpus (Figure 5). No further complications occurred in the three-month follow-up of the patient. 

## Discussion

The heterotopic pancreatic tissue is often seen in the stomach, duodenum, and in the jejunum, Meckel's diverticulum, and mesentery [2]. The incidence of these embryological anomalies is up to 13% in postmortem studies, and the rate of detection after surgery is 0.2% [3]. Two hypotheses related to the formation of heterotopic pancreatic tissue have been proposed; the first hypothesis is that the heterotopic tissue is separated during the rotation of the primitive pancreatic tissue and the second hypothesis is that the ductal tissue belonging to the lateral bud during the development of the pancreatic duct is located abnormally in the embryological period [4].

Patients usually consult with non-specific symptoms, such as epigastric pain, abdominal distension, nausea, and vomiting. Symptoms are thought to be caused by enzymes released from ectopic pancreatic tissue [5]. When we reviewed 16 cases published in the English literature, eight of the 16 patients were operated on the diagnosis of gallbladder stones, three on polyps, and one on acute pancreatitis (Table 1). Both our cases were operated because of the cholecystopathic symptoms associated with the polyp.

**Table 1:** Pancreatic heterotopy cases located in the gallbladder GBS: gallbladder stone

Author	Age (year)	Sex	Preoperative diagnosis	Location in gallbladder	Symptomatic	GBS
Beltran et al. [6]	8	Male	GBS^*^	Body	+	+
Beltran et al. [6]	22	Female	GBS^*^	Neck	+	+
Shiwani et al. [7]	20	Female	GBS^*^	Unknown	+	+
Weppner et al. [8]	26	Female	GBS^*^	Neck	+	-
Elpek et al. [9]	40	Male	Unknown	Neck	+	-
Murakami et al. [10]	49	Female	Polyp	Unknown	-	-
Kondi-Paphiti et al. [11]	58	Female	Polyp	Unknown	+	-
Kondi-Paphiti et al. [11]	48	Female	GBS^*^	Unknown	+	+
Kondi-Paphiti et al. [11]	53	Female	Cancer	Unknown	+	+
Sroczynski et al. [12]	55	Male	Acute pancreatitis	Unknown	+	+
Basrur et al. [13]	40	Female	GBS^*^	Body	+	+
Elhence et al. [14]	18	Female	GBS^*^	Neck	+	+
Lee et al. [15]	36	Male	Adenomyomatosis	Duct	+	-
Kalina et al. [16]	43	Female	GBS^*^	Neck	+	+
Klimis et al. [17]	35	Male	Polyp	Body	+	-
Gucer et al. [18]	80	Male	Sludge	Body	+	-

Heterotopic tissue is frequently localized in the neck and corpus section of the gallbladder, which can be explained by the fact that the lateral pancreas and corpus/neck of the gallbladder are more closely located during embryological development. In our cases, heterotopic tissue was located in the fundus and neck of the gallbladder. It is known that enzymes released from the tissue of the ectopic pancreas may cause neoplastic transformation, but there is no evidence of a gallbladder carcinoma case that has been proven to originate from a heterotopic pancreas in the literature. More extensive series of studies are needed to determine whether enzymes released from the ectopic tissue may cause carcinoma development or whether some of the gallbladder carcinomas are based on enzymes released from the ectopic tissue.

Preoperative radiological examinations are not successful in diagnosis. A definitive diagnosis is made after a histopathologic examination [5]. In our cases, gallbladder polyp and gallstones were detected in ultrasonography, and cholecystectomy was performed for patients having cholecystopathic symptoms. 

## Conclusions

Pancreatic heterotopy located in the gallbladder is an uncommon embryologic anomaly that is rarely seen and a preoperative diagnosis is impossible. This anomaly may cause cholecystopathic symptoms. All the patients presented in the English literature were diagnosed incidentally after cholecystectomy. Heterotopic pancreatic tissue is at risk of having a neoplastic transformation. Therefore, cholecystectomy in the presence of radiologically indicated polyps or nodules in patients with cholecystopathic symptoms without stones in the gallbladder may protect the patient from future cholecystitis, pancreatitis, and malignancy.
